# Come for the looks, stay for the personality? A mixed methods investigation of reacquisition and owner recommendation of Bulldogs, French Bulldogs and Pugs

**DOI:** 10.1371/journal.pone.0237276

**Published:** 2020-08-26

**Authors:** Rowena M. A. Packer, Dan G. O’Neill, Francesca Fletcher, Mark J. Farnworth

**Affiliations:** 1 Royal Veterinary College, Hawkshead Lane, North Mymms, Hertfordshire, United Kingdom; 2 Royal (Dick) School of Veterinary Studies, The University of Edinburgh, Easter Bush Veterinary Centre, Roslin, United Kingdom; 3 School of Animal, Rural and Environmental Sciences, Nottingham Trent University, Nottingham, United Kingdom; Universidade do Porto Instituto de Biologia Molecular e Celular, PORTUGAL

## Abstract

Brachycephalic breeds are proliferating internationally, with dramatic rises in popularity juxtaposed with common and severe breed-related health problems. Physical appearance is as a dominant factor attracting owners to brachycephalic breeds; however, whether these owners will choose their current breed for future ownership and develop ‘breed-loyalty’ in the face of health problems is not yet known. The aims of this study were (1) to quantify levels of, and explore factors associated with, brachycephalic dog owners’ intentions to: (i) reacquire and/or (ii) recommend their current breed to potential first-time dog owners, and (2) to use qualitative methods to explore why brachycephalic dog owners would or would not recommend their current breed. This large mixed methods study reports on 2168 owners of brachycephalic breeds (Pugs: n = 789; French Bulldog: n = 741; Bulldogs: n = 638). Owners were highly likely to want to own their breed again in the future (93.0%) and recommend their breed to other owners (65.5%). Statistical modelling identified that first-time ownership and increased strength of the dog-owner relationship increased the likelihood of reacquisition and/or recommendation. In contrast, an increased number of health problems, positive perception of their dog’s health compared with the rest of their breed, and dog behaviour being worse than expected decreased the likelihood of reacquisition and/or recommendation. Thematic analyses constructed three themes describing why owners recommend their breed: positive behavioural attributes for a companion dog, breed suited to a sedentary lifestyle with limited space, and suitability for households with children. Five themes described why owners recommended against their breed: high prevalence of health problems, expense of ownership, ethical and welfare issues associated with breeding brachycephalic dogs, negative effects upon owner lifestyle and negative behavioural attributes. Understanding how breed-loyalty develops, and whether it can be attenuated, will be key to controlling the current population boom in brachycephalic breeds in the long-term.

## Introduction

In the past decade, the popularity of brachycephalic (flat-faced) dogs has dramatically increased in the UK and internationally [1–3], despite growing scientific evidence and international publicity surrounding the health challenges these breeds face [4]. Brachycephalic breeds are strongly predisposed to a range of disorders intrinsically related to their conformation, including respiratory disease [5, 6], eye disease [7, 8], dystocia [9], spinal disease [10], heat stroke and pneumonia [11], and their lifespan is reduced by 4.1 years compared to dogs with longer muzzles [5]. Indeed, some veterinarians consider Bulldogs, Pugs and French Bulldogs as having “health and welfare too compromised to continue breeding” [12]. Their disease burden is likely to negatively impact animal welfare of brachycephalic dogs [13], and also has the potential to impact human wellbeing, given that owners of pets with chronic illnesses report greater psychological distress and a lower quality of life than owners of healthy pets [14–16]. As such, it is important to understand decision-making around dog breed choice to avoid the future proliferation, or perpetuation, of breeds that are prone to substantial health risks.

Several factors have been identified that affect decision-making of prospective dog owners [17], with several studies highlighting physical appearance as an important consideration for potential owners [18–22]. Physical characteristics that owners consider important include hair type and length [19], body size [20] and iris colour [21]. The external look of a breed (appearance) was recently reported as the factor that most highly influenced a prospective owner to choose a brachycephalic breed. Appearance was significantly more influential for owners of brachycephalic breeds compared with owners of non-brachycephalic breeds, and more influential upon owners of brachycephalic breeds than other factors including breed health and longevity [22]. It is posited that the aesthetic appeal of brachycephalic breeds is driven by the “baby schema effect” whereby brachycephalic face shapes mimic human infantile features to arouse positive emotions and nurturing responses in human adults [23]. Empirical studies have demonstrated that infantile features in cats, dogs and teddy bears increase their attractiveness, and that women show higher ratings for pets with infantile features than men [24]. As well as being biologically rewarding, the attractiveness of the appearance of brachycephalic dogs may be also socially reinforced. A qualitative interview-based study of dog owners categorised owners into two distinct groups based on their motivation for pet ownership: intrinsic or extrinsic [25]. Intrinsically motivated owners prefer to achieve goals that are more innately satisfying and view their pets as unique beings possessing human characteristics that they value for the sake of the individual animal itself. In contrast, extrinsically motivated owners display behaviour that earns social acknowledgment and boosts ‘status’ and see pets as possessions. The authors proposed the latter owner group are more likely to acquire fashionable dog breeds as part of their ‘personal identity project’, commonly those breeds with distinctive physical appearance. A recent Danish study suggested that French Bulldog owners might predominantly fall within this group [26].

Brachycephalic appearance generates an inherent conflict in that their positively perceived physical traits are intrinsically unhealthy, and thus (informed) potential owners must choose whether to prioritise desired aesthetics over health. Evidence suggests that health considerations are generally secondary in the decision to acquire dogs. In a study of American Kennel Club registrations, health and longevity were not correlated with breed popularity, and on the contrary, the most popular breeds tended to have significant health problems [27]. Three quarters of owners adopting dogs from animal shelters reported that a dog’s physical appearance was important (75%), whereas less than half rated the dog’s health as important (49%); however, as these dogs were adopted from animal shelters it is possible their owner’s motivations differ from those purchasing a specific breed [18]. Owners choosing brachycephalic dogs are reported as less influenced by breed health and longevity when compared to owners of non-brachycephalic, popular dog breeds [22]. This same study identified that owners of brachycephalic breeds tended to be younger, first-time purchasers and also first-time owners of dogs [22].

As the popularity of brachycephalic breeds has already reached high levels in the UK, in addition to understanding decision-making about initial acquisition of these breeds, there is increasing need to understand aspects related to re-acquisition from existing owners. A recent study of Danish dog owners reported that nearly one third of French Bulldog owners planned to acquire the same breed in the future, more than owners of any of the other small breeds studied (Chihuahua, Cairn Terrier, Cavalier King Charles Spaniel) [26]. Dog breed-loyalty has received very little scientific attention, but a breed-loyal pet owner has been defined as: “*[An owner with] a positive attitude towards a specific breed of dog*, *[who will] buy that breed when compared with other existing breeds and have a continued allegiance to that breed over long periods of time*.*”* [28]. If current brachycephalic dog owners become breed-loyal then the current ‘boom’ phase of brachycephalic popularity (a phenomenon described in other breeds [27]) may plateau at a high level, rather than dropping back to their pre-boom numbers. Although the effect of external influences on breed popularity, such as dogs in films [29, 30] and dog shows [31] has been explored, the role of owner-owner recommendation has been largely neglected. This could be another important route to increasing breed popularity, especially if peers promote positive traits in a particular breed. The aims of this mixed-methods study were twofold:

1. To use quantitative methods to quantify levels of, and explore factors associated with, brachycephalic dog owners’ intentions to: (i) reacquire and/or (ii) recommend their current breed to other future owners
2. To use qualitative methods to explore reasons why brachycephalic dog owners would or would not recommend their breed to other owners

## Methods

### Participants

The population sampled for this study was described in detail in a previous publication [32]. Briefly, current owners aged >18 years old of the three most commonly registered Kennel Club (KC) brachycephalic breeds (French Bulldog; Pug; Bulldog) were purposively sampled via online forums and social media platforms in June and July 2017. These breeds were selected from amongst those considered brachycephalic due to the current high levels of popularity they have reached, preceded by a marked rapid expansion in popularity, and as such results are unlikely to be generalisable to all brachycephalic breeds. Participants with more than one dog meeting the inclusion criteria were requested to answer the survey with regard to the most recently acquired dog. Respondents were informed that submission of the survey would indicate consent to usage of their data for research purposes. The survey was approved by the Human Ethical Review Committee (HERC) at the R(D)SVS, University of Edinburgh.

### Survey structure

Sections 1–6 of this survey are reported in an existing publication [32] and details are included in S1 File. Briefly, this survey explored (1) owner and dog demographics, (2) veterinary history of their dog, (3) presence and severity of airway dysfunction, (4) owner perception of health problems in their dog, (5) owner expectations for veterinary costs, exercise levels, maintenance levels and behaviour, and (6) the dog-owner bond, as quantified by the Monash Dog-Owner Relationship Scale (MDORS) [33].

Section 7 is the focus of the current study and explored re-acquisition desire and peer-peer recommendation of an owner’s current breed. Owners were asked to report whether they would choose to own their current breed again (Yes/No). Although a previous study [26] posed a similar question on plans to procure a new dog in the future, with five possible response options (“have no plan to”, “don’t know”, “yes, but not the same breed”, “yes, maybe the same breed”, and “yes, for sure the same breed”; those authors simplified these values to a binomial variable for logistic regression (0 = “no for sure”; 1 = “yes for sure”). A such, we elected to use this variable format in our study from the outset. In addition, owners were asked whether they would recommend their breed to a first-time dog owner (Yes/No). They were then asked to report any features of their breed that they *would* recommend, and aspects of their breed that they *would not* recommend. Owners were provided with a free text box to give their response to the latter question.

### Quantitative analysis

Following initial cleaning of data in Microsoft Excel 2013, statistical analyses were carried out in IBM SPSS Statistics v24 (SPSS Inc, Chicago, IL, USA). Binary logistic regression models were used to determine which factors predicted owner’s desire to acquire another dog of the same breed, or to recommend their breed to a potential first-time dog owner. Binary outcome variables tested were whether owners would re-acquire (Yes/No) and recommend (Yes/No) their current breed. Variables tested for their association with owner attitudes towards reacquisition or recommendation of their current breed were: canine demographics (breed, age); owner demographics (age, gender, whether they were a first-time dog owner (1/0), children in household (1/0), country; veterinary experiences (veterinary costs per year (£); veterinary costs to date (£); number of conformation-related surgeries); health scores and owner perceptions of their dog’s health (owner reported breathing score (ORB); heat intolerance score; eating difficulty score; disordered sleeping score; number of perceived health problems; health compared to the rest of their dog’s breed; overall health rating); owner expectations of the breed vs. reality of ownership (veterinary costs, exercise levelsand maintenance levels: scored as less than expected, met expectations or more than expected, and overall behaviour: scored as worse than expected, met expectations, or better than expected) and the dog-owner bond as measured by the MDORS (emotional closeness, perceived costs, dog-owner interactions). Factors with liberal associations in univariable tests (*P* < 0.2 in logistic regression) were taken forward for multivariable evaluation. Model development used manual backwards stepwise elimination. The Hosmer-Lemeshow test statistic was used to evaluate model fit and graphical analysis of residuals. Biologically meaningful pairwise interactions were evaluated and retained in the final models if significant. Results are reported as mean ± standard deviation [SD] for normally distributed variables, and median [IQR] for non-normally distributed data. A p value of < 0.05 was considered significant.

### Qualitative analysis

Free-text responses were analysed using thematic analysis methods described by Braun and Clarke [34]. Data were initially separated into those pertaining to characteristics owners *would* recommend or *would not* recommend about their breed. Within this, an inductive approach was employed with coding and theme development driven by the content of the comments. One author (RMAP) familiarised herself with the data by reading all the free-text responses. This was followed by categorisation of free-text data into latent and semantic codes. From these codes, subthemes and themes were actively constructed. Due to the nature of data collection, analysis was performed after all of the data were collected. Although not a standard element of the thematic analysis process, due to the large scale but relatively shallow level of the data collected, theme and sub-theme frequencies were measured to give an indication of their prominence, as has been used in other studies utilising large numbers of free-text responses [35]. However, as is standard in qualitative research, statistical analyses were not performed on these data [36].

## Results

### Demographics

In total, 2168 valid responses were received from owners of Pugs (n = 789), French Bulldogs (n = 741) and Bulldogs (n = 638). Respondents were predominantly from the UK (72.0%) followed by USA (13.9%) and Canada (2.4%). The majority of respondents were aged 25–34 years (34.2%) and female (89.1%). The median age of study dogs was 2.17 years (IQR: 0.92–4.33), 58.4% of dogs were male and 42.0% of all animals were entire. Further details, including breakdowns by breed are reported in Packer et al, 2019 [32].

### Attitudes to reacquisition and recommendation

#### Reacquisition desire

The majority of owners (93.0%, n = 2012) would choose to own their current breed again in the future, with no difference identified in levels of reacquisition desire between the three breeds. At the univariable level, 19 variables were significantly associated with whether an owner would reacquire their breed (S1 File). Seven variables remained in the final multivariable model (Table 1). First-time dog owners had over two times the odds of reacquisition desire compared to owners who had owned dogs before (Odds Ratio [OR] 2.45, 95% Confidence Interval [CI_95]_ 1.49 to 4.03). The odds of reacquisition desire decreased with increasing number of owner-recognised BOAS-related health problems (Brachycephalic Obstructive Airway Syndrome; p<0.001). Owners of dogs considered to have health less than the best possible (worst possible, very poor, moderately poor, good) had a reduced odds of reacquisition desire compared to owners who considered their dogs to have the best possible health. Owners who considered their dog to be much healthier than average for the breed had reduced odds of reacquisition desire (OR 0.36, CI_95_ 0.19 to 0.69) compared to those considered to have average health. Owners whose dogs behaved worse than they expected had a reduced odds of reacquisition desire (OR 0.60, CI_95_ 0.37 to 0.97) than those whose behaviour met their expectations. An increased level of emotional closeness and a reduced perception of the costs of ownership (as indicated by a Higher Perceived costs value) were associated with an increased odds of reacquisition desire (p<0.001).

**Table 1:** Results of multivariable binary logistic regression for risk factors associated with owner desire to reacquire their current breed.

Variable	Sub-category	OR (95% CI)	P
First-time dog owner	Yes	2.45 (1.49–4.03)	<0.001
	No	*Reference*	
No. of BOAS-related health problems	-	0.54 (0.45–0.66)	<0.001
Compared to the rest of their breed	Much less healthy	0.70 (0.21–2.35)	0.568
	Less healthy	0.64 (0.32–1.30)	0.217
	Average health	*Reference*	
	Healthier	0.84 (0.50–1.42)	0.511
	Much healthier	0.36 (0.19–0.69)	0.002
Overall health rating	Worst possible	0.05 (0.01–0.44)	0.008
	Very poor	0.07 (0.02–0.22)	<0.001
	Moderately poor	0.17 (0.07–0.45)	<0.001
	Good	0.19 (0.08–0.43)	<0.001
	Moderately good	0.28 (0.13–0.61)	0.002
	Very good	0.53 (0.27–1.04)	0.064
	Best possible	*Reference*	
Overall behaviour	Better	1.25 (0.72–2.15)	0.430
	Met	*Reference*	
	Worse	0.60 (0.37–0.97)	0.037
Emotional closeness	-	2.55 (1.89–3.44)	<0.001
Perceived costs	-	2.17 (1.59–2.97)	<0.001

N.B. For the variable ‘Perceived costs’, a higher score indicates a lower perceived cost of ownership.

#### Recommendation–quantitative analysis

Two thirds of owners (65.5%, n = 1275) would recommend their current breed to a potential first-time dog owner, with significant differences at the univariable level between the three breeds; highest in the Pug (73.7%) and lower in the Bulldog (56.2%; OR 0.46 (CI_95_ 0.36–0.58), p<0.001) and French Bulldog (64.6%; OR 0.65 (CI_95_ 0.52–0.82), p<0.001). At the univariable level, 20 variables were significantly associated with whether an owner would recommend their breed to a potential first-time dog owner (S1 File). Nine variables remained in the final multivariable model (Table 2). Bulldog and French Bulldog owners had decreased odds of recommending their breed (OR 0.50 and 0.64. respectively) compared to Pug owners. First-time dog owners had over three times the odds of recommending their breed compared to owners who had ever owned dogs before (OR 3.09, CI_95_ 2.38 to 4.01). The odds of recommending their current breed decreased with increasing number of conformation-related surgeries (p<0.001) and owner-recognised BOAS-related health problems (p<0.001). Owners of dogs considered to have health less than the best possible (very poor, moderately poor, good, very good) had reduced odds of recommending their breed compared to owners who considered their dogs to have the best possible health. Owners who considered their dog to be healthier or much healthier than average for the breed had reduced odds (OR 0.64 and 0.46, respectively) of recommending their breed compared to those considered to have average health. Owners whose dogs behaved worse than they expected had reduced odds of recommending their breed (OR 0.59, CI_95_ 0.43 to 0.80) than those whose behaviour met their expectations. An increased level of emotional closeness (p = 0.006) and a reduced perception of the costs of ownership (as indicated by a higher Perceived Costs value) (p = 0.034) were associated with increased odds of recommending their breed.

**Table 2:** Results of multivariable binary logistic regression for risk factors associated with owner desire to recommend their current breed to a potential first-time owner.

Variable	Sub-category	OR (95% CI)	P
Breed	Bulldog	0.50 (0.38–0.64)	<0.001
	French Bulldog	0.64 (0.49–0.83)	0.001
	Pug	*Reference*	
First-time dog owner	Yes	3.09 (2.38–4.01)	<0.001
	No	*Reference*	
No. of conformation- related surgeries	-	0.75 (0.66–0.87)	<0.001
No. of BOAS-related health problems	-	0.74 (0.65–0.85)	<0.002
Compared to the rest of their breed	Much less healthy	0.49 (0.18–1.38)	0.178
	Less healthy	0.79 (0.45–1.38)	0.400
	Average health	*Reference*	
	Healthier	0.64 (0.49–0.84)	0.001
	Much healthier	0.46 (0.33–0.64)	<0.001
Overall health rating	Worst possible	3.70 (0.26–51.91)	0.332
	Very poor	0.23 (0.09–0.58)	0.002
	Moderately poor	0.29 (0.15–0.53)	<0.001
	Good	0.33 (0.21–0.52)	<0.001
	Moderately good	0.40 (0.28–0.60)	<0.001
	Very good	0.59 (0.45–0.78)	<0.001
	Best possible	*Reference*	
Expectation of overall behaviour	Better	1.24 (0.94–1.62)	0.124
	Met	*Reference*	
	Worse	0.59 (0.43–0.80)	0.001
Emotional closeness	-	1.29 (1.08–1.54)	0.006
Perceived costs	-	1.25 (1.02–1.53)	0.034

N.B. For the variable ‘Perceived costs’, a higher score indicates a lower perceived cost of ownership.

#### Recommendation–qualitative analysis

Eight themes were constructed from the free text analysis: three related to characteristics of a breed which owners *would* recommend and five related to characteristics owners *would not* recommend. The following text summaries describe each theme and sub-theme and are accompanied by illustrative quotes from owners involved in the study.

#### Themes related to features owners *would* recommend

*Theme 1*: *Positive behavioural attributes for a companion dog*. Positive behavioural traits were the most dominant theme amongst owners’ recommendations for their breed. This theme incorporated several sub-themes pertaining to specific valued elements of their dog’s behaviour. These painted a picture of dogs that fulfilled a strong companion role that inspired substantial breed-loyalty from their owners; “*I would never have any other breed*. *They are fun loving*, *gentle*, *loyal babies*” (Bulldog, O1984).

*Subtheme 1*.*1*: *A loving*, *loyal companion*. Owners placed a strong focus on how loving their dog’s breed was (n = 422, 31.6% comments), and was considered by many owners to be the most loving breed they had experienced. Owners often considered this to be a unique trait to their breed; however, this was observed across all three breeds. “*They are the most loving breed*” (O261, Bulldog); “*The most affectionate dogs I have ever encountered*” (French Bulldog, O61); “*Very affectionate loving companion”* (0684, Pug).

There was an emphasis on physical affection between dogs and their owners, with owners valuing dogs who appeared to enjoy being cuddled and played with; “*All aspects of the dogs’ behaviour are fantastic*, *they are loving*, *affectionate*, *love being cuddled*” (O389, French Bulldog); “*He is a sweet kind natured boy that just wants to cuddle and play*” (French Bulldog, 02136); “*They love being held like babies and getting belly rubs*” (Pug, 0186). Alongside an affectionate nature towards their owners, loyalty also appeared an important behavioural trait to owners (n = 259, 19.4% comments), which was seen in all three breeds, although owner descriptions of what they meant by loyalty were lacking; “*Very loyal to their person or people*” (O349, Bulldog).

*Subtheme 1*.*2*: *Comical*, *clown-like breed*. Humour and a comical nature were common behavioural traits upon which many owners recommended their breed (n = 175, 13.1%). Owners enjoyed observing their dog’s humorous personalities and viewed their dog as a source of entertainment in the household; *“I believe they are the most fascinating dogs*, *their little personalities are hilarious*. *I've never laughed so much with my dog*.*”* (O397, Pug); *“He has a very clownish personality that keeps us entertained”* (O743, Pug). Owners commonly described their dog’s as clown-like, with some owners indicating their belief that their dogs intentionally acted in a comedic manner; *“They are funny little clowns*. *I think they know they are funny”* (O897, French Bulldog).

*Subtheme 1*.*3*: *Playful nature*. The willingness of these breeds to engage in play with their owners was commonly cited (n = 100, 7.5%) as a positive behavioural attribute. This was often viewed by owners as part of their personality; “*Very playful and a character which brings joy to a home*” (Bulldog, O1424); “*Personality is amazing*. *They're nuts and so playful*” (Pug, O1308).

*Subtheme 1*.*4*: *Trainability*. Ease of training was described by some owners as a positive trait of their breed (n = 67, 5.0%), although this was sometimes given with the caveat that their dog exhibited other undesirable behaviour traits that hampered training attempts, such as stubbornness, which was also a sub-theme within responses to why owners would not recommend their breed; “*Although he's very stubborn he's excellent at training and understanding what you ask of him*” (Bulldog, O1395). Several owners emphasised the importance of using appropriate training techniques, “*If trained correctly with positive training they are very intelligent and learn quickly*” (French Bulldog, O401) and that the intelligence of this breed is potentially underestimated “*Way more intelligent than they are given credit for*” (Pug O1046).

*Theme 2*: *Breed suited to a sedentary lifestyle with limited space*. Many owners focused on the suitability of their breed for the owners’ lifestyle, including the size of their living space and activity levels. A strong focus was placed on how convenient and low maintenance Bulldogs, French Bulldogs and Pugs were for the (often sedentary) lifestyles of owners.

*Subtheme 2*.*1*: *Low exercise requirements*. Perceptions of low exercise requirements combined with ‘lazy’ temperaments were strongly positively promoted by some owners (n = 74, 5.5%), traits believed to make them ideal dogs for owners unable to provide moderate to high levels of exercise for their dog for a variety of reasons; “*Pugs are good for low*, *low energy people*, *disabled people*, *elderly*, *or apartment living*.” (O1493, Pug); “*Lazy and not needing tons of exercise makes them a good dog for working people*” (O230, Bulldog). Others indicated that their breed would suit owners who wanted a dog, but did not want to exercise them; “*Requires small amounts of exercise*, *so someone not overly keen on walking would suit this breed well*” (O1248, French Bulldog). Some owners tried to explain these perceived differences based on the ‘purpose’ these breeds were selectively bred for, highlighting their differences to other dog breeds; “*Bulldogs do not require as much "dog-centric" tasks as other dogs*. *They do not need much exercise*. *They were not breed (sic) for a job*, *so they are happy to be companions*. *They are sweet and easy*.” (O871, Bulldog).

*Subtheme 2*.*2*: *Low space requirements*. Perceptions of low space requirements were promoted by some owners (n = 41, 3.1%), which they believed enabled these breeds to live in households unsuited to larger dogs; “*Small and manageable for living in a condo*” (French Bulldog, O129). This was sometimes coupled with their perceived low exercise requirements, with owners portraying a dog that is easily accommodated into many human lifestyles; “*Don't need a huge amount of space*, *or a huge amount of exercise*” (O101, French Bulldog).

*Theme 3*: *Suitable for households with children*. Positive relationships with children (n = 157; 11.8%) were promoted as a positive breed trait for many owners, with some owners describing a quiet, calming nature, while others described a fun, playful nature that children responded well too; “*These dogs are wonderful with children*. *We have ages newborn to 12 and he is the perfect loving dog*” (O1265, French Bulldog); “*She's very good with the children and loves to run and play with them*” (O2106, Bulldog). Several Pug owners pointed towards their breeds’ perceived abilities to interact with neurodiverse children in a positive manner; “*They are brilliant with children too*, *and in my experience they are brilliant with children with autism*, *ADHD and sensory processing issues as they are so laid back and calming*” (0636, Pug); “*Child friendly companion nature*, *especially to those less able or children with autism*” (O1199; Pug); “*Very forgiving (for example with small children)*, *sensitive to humans' moods and emotional needs (great support for our autistic child)*” (O484, Pug).

#### Themes related to features owners would not recommend

*Theme 4*: *High prevalence of health problems*. Health problems were a common reason why owners would not recommend their breed to prospective owners, including (by frequency) overheating (n = 108, 8.1%), breathing problems (n = 95; 7.1%), skin and allergy problems (n = 93, 7.0%) and eye problems (n = 30, 2.2%). Many owners were concerned that a first-time owner of the breed would be unaware of the level of healthcare these breeds may need, and that a lack of understanding may lead to poor welfare for any dogs they acquired; “*I would suggest first-time dog owners be cautious*, *as they may not be aware of how much care they need*. *i*.*e*. *they do not need as much exercise as some other breeds*, *and could have breathing issues which would need to be looked after carefully*” (French Bulldog, O563); “*I don't think a first-time owner would realise*, *or act quick enough to an eye injur*y” (Pug 1167).

Despite acknowledging health issues, many owners indicated that they would not change their own dog and would reacquire their breed in the future. Some owners justified their own dog’s health problems by explaining that the positive elements of their breed outweighed the negatives; “*I love my Pug and wouldn't change him for the world but can see that their breathing issues aren't fair*.” (Pug, O1655); “*He is just perfect and despite some health issues we wouldn't change him for the world*!” (French Bulldog, O1495). Owners were often keen to emphasise that their own dog was not in as poor health as other members of their breed; “*I have kept my Pug lean and slim so I have had less problems than the average owner*, *but the short face is an issue with overheating*. *My Pug’s face is not as bad as others but can still cause an issue if we are not careful*.” (Pug, O546); and that owners should be cautious of acquiring one of the many members of their breed with health issues; “*I wouldn't recommend the possible breathing issues to a first-time dog owner*. *My Pugs are very healthy*, *but I've seen lots that aren't*” (Pug, O509).

Health problems were sometimes a divisive issue between owners and non-owners of brachycephalic breeds, with perceptions of unfounded biases against their breeds. Some owners defended their breed’s health problems and considered them as comparable to those of other breeds; “*I find my Pug an affectionate lovable little guy who we wouldn’t be without*, *the only time he struggles is in extreme heat but he is black so we just keep him in the shade with plenty of fluids*. *I would quite happily buy another Pug and would not be put off by all the negativity around this breed as all other breeds have their own problems*” (Pug, O1453). Owner perceived ‘negativity’ regarding the health of their breed also extended to vets, with vet-owner conflict due to diverging views on health; “*Pugs get a lot of negative attention for breathing difficulties and some vets are biased when it comes to these breeds*. *I changed vets because our first vet said he needed surgery for his nostrils*. *Since changing our new vet who is experienced with pugs have never mentioned it*” (Pug, O428).

*Theme 5*: *Expense of ownership*. For some owners, the costs of owning their breed had been considerable, particularly those related to healthcare (n = 111, 8.5%); “*Aspects I'd not recommend—they're expensive to insure and with regards [to] vet bills when something inevitably goes wrong*” (Pug, O884). Some comments made it clear that the expense of ownership was unexpectedly high; “*Bulldogs look sturdy*, *but they are delicate flowers*. *It's always something*, *and the vet bills are way more than I expected*” (Bulldog, O1146). Owners told cautionary tales of financial difficulties if potential owners were financially ill-prepared for ‘inevitable’ veterinary bills; “*If you do not have money saved up*, *or a decent paying job this breed can and will bankrupt you*.” (French Bulldog, O1907). Several owners highlighted the importance of insurance in saving money on vet’s bills; “*If one plans to add a French Bulldog to their family (because you don't just own one of these dogs)*, *it is imperative to have pet insurance*. *Even with insurance*, *we have spent exorbitant amounts of money on her health*.” (French Bulldog, O710).

*Theme 6*: *Ethical and welfare issues associated with breeding brachycephalic dogs*. Some owners expressed concerns over the ethical and welfare implications of breeding brachycephalic dogs, including the welfare costs to individual dogs, but also more broadly a desire to not support inhumane breeding practices.

*Theme 6*.*1*: *Concerns over the welfare costs of poor breeding to the individual dog*. A small number of owners (n = 29, 2.2%) recommended against their breed due to the welfare impairments incurred to individual dogs due to their breeding, which some owners described as suffering. Even in the face of other positive traits, some owners considered these impairments unjustifiable and potentially avoidable: “*I adore Pugs and their bright*, *engaged personalities*, *and I am saddened that the physical traits they are bred for create so many health problems for them*, *essentially maiming them*. *They are such sweet dogs*, *and this fact makes me sad for them*. *I don't want to perpetuate a destructive standard that makes their lives difficult*, *so I am hesitant about seeking out another Pug simply because I don't want an animal to suffer and struggle*.” (Pug, O1842).

*Theme 6*.*2*: *Concerns over the encouragement of bad breeding practices*. Broader concerns regarding breeding practices used to produce their current breed were cited by a minority of owners as reasons against recommending their breed (n = 44, 3.3%), including inbreeding, ‘overbreeding’ and breeding without due regard for health; “*Currently*, *French Bulldogs*, *for the large part*, *have too many breeder-caused health issues to be bred any longer and sold*. *It is unethical and cruel*. *So*, *I would not recommend the breed to anyone*.” (French Bulldog, O191). Other owners were concerned about the type of people attracted to supplying brachycephalic puppies in the current popularity boom, including backyard breeders and illegal imports; “*As we know they are becoming popular and are expensive to buy*, *this is not attracting the right people to A buy and B breed*. *I recommend them because they are great family dogs*, *but because of the horrible breeding I wouldn't for their health problems*” (Bulldog, O1298).

Some owners were reticent to recommend their own dog’s breed until changes to breeding practices had been made; “I *would not recommend a Pug to anyone until we have started to address their health problems*, *I am lucky with mine but I still recognise that he cannot breathe as easy as a Labrador*.” (Pug, O959), and advocated educating potential owners regarding health problems prior to acquisition; “*I would NOT recommend this dog due to their health problems*. *Specifically breathing*. *We need to be breeding dogs who don't suffer and struggle to breathe and we need to be honest to potential owners about these issues before they buy them*” (French Bulldog, O2138).

*Theme 7*: *Negative impacts upon owner lifestyle*. Although some physical and behavioural traits of Bulldogs, French Bulldogs and Pugs were considered beneficial to owner lifestyles, others were considered annoying and burdensome, and to be avoided.

*Subtheme 7*.*1*: *High maintenance requirements*. The high maintenance requirements needed to manage existing health issues or prevent future health issues were considered a key reason to recommend against breeds by many owners (n = 219, 16.4%); “*He's got a great personality*, *but would not recommend if you don't plan on grooming often i*.*e*. *wiping skin folds*, *cleaning ears*, *trimming nails etc*.” (French Bulldog O796). Comparisons to the care needed to look after young children or babies were made to emphasise this point, with owners requiring time and patience to give this level of daily care; “*They require care and attention on par with a well-behaved toddler*. *This is not what most people expect or want in a dog*, *so I do not encourage people to buy this breed*.” (Bulldog, O2021).

*Subtheme 7*.*2*: *Shedding*. Annoyance over the additional household cleaning necessitated from shedding hair was expressed by owners of all three breeds (n = 71, 5.3%) who often did not anticipate this as a problem before acquiring their dog; “*An annoying aspect of the breed is that they fart and they lose a lot of fur*, *so I wouldn't recommend this breed to people who are intolerant of such actions and to people that don't like to clean very often*” (French Bulldog, O1525).

*Subtheme 7*.*3*: *Increased respiratory noise*. Annoyance was expressed over respiratory noise some dogs produced while both awake and asleep (n = 17, 1.3%), but largely focused on snoring at night leading to disrupted sleep for the owner; “*Pugs snore very very loudly this can cause sleep disruption*, *I would make potential owners aware*” (Pug, O1497). Respiratory noise was described in a variety of situations including while chewing toys and eating, which some owners found annoying; “*Oh*, *and of course they snore a lot*, *so*, *if anyone is annoyed by snoring*, *choose not a flat nose breed*." (French Bulldog, 01525). Comments regarding snoring were often mentioned independently of references to airway disease, discussed as if an inherent feature of the breed, and sometimes viewed as a humorous or even valued trait; “*There* (sic) *snoring is horrendous lol* [laugh out loud]” (Bulldog, O1975); “*He grunts whilst breathing (means we never lose him on walks) but he snores non stop when asleep*” (Bulldog O941).

*Theme 8 Negative behavioural attributes*. Owners described several elements of their dog’s behaviour that made them advise against their breed, ranging from annoying to potentially dangerous traits, some of which were unexpected of the breeds studied.

*Subtheme 8*.*1*: *Training challenges and stubborn nature*. Despite some owners citing ease of training as a valuable trait upon which to recommend their breed, many others (n = 177, 13.2%) experienced challenges in attempts to train their dog, which many explained was due to their dog’s stubbornness. This was observed across all three breeds; “*Training is harder due to their little stubborn personalities*” (Pug, 0641); “*Stubborn breed to train*. *Can test your patience to the limit when young and boisterous*” (Bulldog, O925); “*They’re stubborn*. *Training needs to be a hard daily thing*” (French Bulldog, O1176).

*Subtheme 8*.*2*: *Neediness and separation related behaviours*. Owners commonly portrayed intense dog-owner relationships characterised by dogs incapable of being left alone (n = 139, 10.4%), some for even short periods of time; “*The only reason I wouldn't recommend the French Bulldog is because they are very needy and don't like being left alone at all*” (French Bulldog, O494); “*Very needy so if you can't be with them 16 out of the 24hrs you probably shouldn't have this breed*” (Pug, O916). Several separation related behaviours were reported by owners including destructive behaviour, elimination and vocalisation; “*Not the best dog to leave alone*. *If you do leave them alone be prepared to come home to something chewed or come home to a mess*” (Bulldog, O782).

*Subtheme 8*.*3*: *Aggressive tendencies*. Although rarely reported (n = 30; 2.2%), some owners reported problems with aggressive tendencies in their dog, including resource guarding, and aggression towards both people and other dogs. Some owners felt this potential aspect of their breed was not well known outside of their breed community and was unexpected; “*No one advertises it*, *but French Bulldogs can be aggressive to humans and other dogs*. *After being on a Facebook French Bulldog group I've came (sic) to realise I am far from alone with this problem*” (French Bulldog, O766).

## Discussion

This is the first large-scale study to explore owners’ desires to reacquire or recommend the most popular brachycephalic breeds in the UK. Inference from the results is strongly enhanced by the application of the mixed methods approach which builds on the quantitative statistical analyses of the data with a qualitative approach. This elicits deeper emotional and perceptual insights from these owners and yields previously uncovered perspectives. The majority of owners indicated they would reacquire their breed in the future (93.0%), and around two-thirds of owners (65.5%) would recommend their breed. Reacquisition desire did not significantly differ between the three breeds; however, Pug owners were more likely to recommend their breed than Bulldog and French Bulldog owners. The high levels of both reacquisition desire and recommendation are of concern as this may indicate that the current spike in brachycephalic popularity [1–3] is likely to persist, due to the development of breed-loyalty [28]. The findings from this study may inform future efforts to reduce levels of ownership of these breeds by highlighting (i) undesirable traits (as identified by current owners) about which potential owners can be educated, and (ii) desirable traits that may be found in other dog breeds, to promote as alternatives to potential or current owners of brachycephalic breeds.

### Influence of dog behaviour

Dog behaviour was a key influence on an owner’s desire to reacquire or recommend their current breed. Owners whose dogs behaved worse than they expected had reduced odds of reacquisition desire or recommending their breed, while positive behavioural characteristics were core reasons *for* recommendation, and negative behavioural traits *against* recommendation of their breed. Previous work indicates that Pugs, Bulldogs and French Bulldogs are partly acquired based on perceived positive behavioural traits, namely making good companion dogs, and being good breeds for households with children [22]. Breed standards of the three brachycephalic breeds studied here describe dogs with an affectionate, happy nature [37], and there is some previously published empirical evidence supporting these positive behavioural traits. Dogs with higher cephalic indices (shorter muzzles) are reported as more affectionate, cooperative and interactive with unfamiliar humans than dogs with lower cephalic indices (longer muzzles) in both owner-reported and practical tests of dog behaviour [38, 39]. Dog-owner communication may be enhanced in brachycephalic dogs; with greater performance at utilising the human point gesture compared to dolichocephalic breeds [40], and brachycephalic dogs displaying the longest looking times at human and dog portraits compared with mesocephalic breeds (with a skull of medium proportions e.g. Labrador Retriever) and doliocephalic breeds (with a relatively long skull e.g. Greyhound) [41]. Rather than preference being purely based on their paedomorphic (baby-like) appearance, this may support the suggestion that the popularity of these breeds is also because humans might have preferred animals that looked at them for longer durations because this enhanced the effectiveness of communicative interaction [42]. Given experience plays an important role in the development of behaviour, including dog-owner interactions e.g. [43–45], it is also possible that brachycephalic breeds are not intrinsically predisposed to behave differently to dogs with longer muzzles; but that owners consciously or unconsciously treat them differently, which results in differing reports in observed behaviours.

In contrast to the positive behavioural traits reported here, there is existing evidence for negative behavioural traits in brachycephalic dogs which may defy owner expectations and reduce their likelihood of wanting to own them again or recommend to others. Compared to longer muzzled breeds, brachycephaly was associated with higher levels of persistent barking and compulsive staring in an owner-reported survey [39], and with higher levels of chase proneness and aggression and in practical assessments of dog behaviour [46]. Brachycephalic (and dolichocephalic) breeds have been perceived to be less trainable than mesocephalic dogs [47], and aggression was the 13^th^ most common reason French Bulldog’s were presented to UK veterinary surgeons in 2013 [48]. As such, presenting a balanced view of brachycephalic behaviour is key to education initiatives around choosing suitable breeds, highlighting that within-breed differences in behaviour are likely, influenced by genetics but also environment and experience [49].

Perceived suitability for living with children was also a core reason for recommendation by Bulldog, French Bulldog and Pug owners and may be a key factor perpetuating brachycephalic popularity in young families. In international studies of the ‘ideal companion dog’, safety with children was the most desirable behavioural characteristic, with over two thirds of Australian [19] and three quarters of Italian [20] owners considering it an ‘extremely important’ trait. With no conclusive evidence that brachycephalic breeds are safer breeds for households with children, and indeed, no robust evidence that breed is a risk or protective factor for dog bites [50], recommending brachycephalic dogs on this basis is unreliable and potentially irresponsible.

### Influence of owner lifestyle

Owner lifestyle, and perception of a breed’s ability to fit within it, was influential in explaining both why an owner would and would not recommend their breed. Bulldogs, French Bulldogs and Pugs were promoted by owners as requiring little exercise or space, deeming them suitable for busy lifestyles, city-living and households considered unsuitable to other larger or more active breeds. These findings are in agreement with previous studies that identified brachycephalic breeds as being more likely to live in apartments than non-brachycephalic breeds, and that breed size being suited to an owner’s lifestyle was more influential in the decision-making on acquisition of brachycephalic than non-brachycephalic breeds [22]. Trends in canine population morphology in Australia indicate that dogs with flatter faces, and shorter and smaller breeds have become relatively more popular [2]. Combined with many owners desiring a ‘lazy’ low energy dog, it appears that some brachycephalic breeds occupy an ownership niche, in which traditional ownership activities such as dog walking are not highly valued, despite being one of the most common reasons (behind companionship) for acquiring dogs more generally [51]. This is potentially problematic for both dog and owner; owners may miss out on the benefits of dog walking for physical and social health [52], and dogs may, despite preconceived laziness, require a greater level of exercise than they are provisioned, and not have their physical and mental needs met. Preconceptions of breed ‘laziness’ are inherently problematic. If individuals truly have a reduced motivation for exercise, this may be driven by pain or disease, for example osteoarthritis [53], or reduced exercise tolerance associated with airway obstruction common in brachycephalic breeds [54], which may go unrecognised and undertreated. If dogs are motivated to exercise but owners do not recognise this and consequently under-exercise them, dogs may be at risk of becoming overweight or obese [55], a commonly recognised problem in brachycephalic breeds such as the Pug [56]. However, promoting exercise in brachycephalic dogs poses a dilemma; BOAS is prevalent in the breeds studied here, and exercise could put some dogs at increased risk of some significant illnesses or even death. Brachycephaly impairs thermoregulation [57] and brachycephalic breeds have recently been reported with over twice the odds of heat-related illness compared to mesocephalic dogs, which is often precipitated by physical exertion [58]. A study of Pugs and French Bulldogs (n = 100) found that 87.0% of owners considered their dog as exercise intolerant, with 70% of dogs mainly affected in the summer [54]. Half of owners (50%) reported deterioration of respiratory signs began at temperatures even below 20°C. This background of predisposition to heat-related illness should be emphasised to prospective owners considering acquiring these breeds. Prior awareness of these risk may deter ownership, particularly those who are risk averse [59] or value recreational dog walking [60]); given that even mild temperatures could lead to significant compromise in health and welfare, and in the summer could result in death. In the UK, the Brachycephalic Working Group in currently engaged in a welfare campaign to encourage prospective dog owners to ‘Stop and think before buying a flat-faced dog’ [61].

Several characteristics were considered to negatively impact upon owner lifestyle, leading to recommendations against brachycephalic breeds. Education about these characteristics to potential brachycephalic owners who are otherwise not influenced by messaging regarding breed health from an ethical perspective, may encourage them to consider other breeds instead. High levels of coat shedding were considered undesirable and were often unexpected by brachycephalic dog owners. The desirability of non-shedding dogs is exemplified in the international population boom in ‘designer cross breeds’ that include Poodle genetics (e.g. Labradoodle, Cockapoo) [62], with such crosses being marketed as low-shedding and hypoallergenic, despite limited evidence for reduced allergen production by these dogs [63]. Negative impacts of health problems upon owner lifestyle included increased and abnormal respiratory noise, particularly snoring, interruption of owner sleep or annoyance during the day. Comments often reflected a lack of understanding regarding the underlying cause of these noises, which were commonly disconnected from any concern regarding breed health, and instead considered normal for their breed. In some cases, snoring was considered funny, similarly to other publicly misunderstood canine pathologies that have been viewed in this way [64]. Finally, some owners considered the high maintenance requirements of their dog as undesirable. Although caregiving may be the catalyst of the dog-owner bond for some owners and a valued facet of their relationship [65], others viewed their maintenance needs as excessive. This population of brachycephalic dog owners who did not expect or desire this level of maintenance may be at risk of abandoning their dogs; indeed, relinquishments of brachycephalic dogs with health problems to rescue centres has recently surged in the UK [66].

### Influence of health

The health of an owner’s current dog influenced the odds of reacquisition desire and of recommending their breed. Owners who reported that their dog had a greater number of BOAS-related health problems, or considered their dog to have health less than ‘the best possible’ had reduced odds of wanting to own their breed again or recommending it to others, compared to those with better health. In addition, those owners whose dogs had undergone a higher number of conformation-related surgeries had decreased odds of recommending their breed, compared to those who had undergone fewer. Studies indicate that owners of pets with chronic illnesses report a greater ‘caregiver burden’, psychological distress and reduced quality of life compared with owners of healthy pets [14–16], and thus owners may want to protect their mental health and avoid this in future pets, and not wish these experiences upon others. Many of the common breed-related disorders in brachycephalic dogs are either chronic e.g. airway disease [5, 6], or recurring e.g. corneal ulcers [7, 8] and may both be emotionally and financially burdensome for owners. Although these were significant effects, it should be noted that many dogs in this study population had significant health problems [32], yet the vast majority of owners would still elect to own their breed again, and thus these effects are not as strong as might be expected. Health has previously been found to impact re-acquisition decisions in the French Bulldog. In a study of Danish dog owners, 29.2% of French Bulldog owners (n = 185) indicated they were certain they would acquire the same breed again, 28.1% would consider the same breed, and 10.3% would not acquire the same breed again. The remaining third either had no plans to acquire another dog (5.4%) or were unsure if they would acquire another dog (27.0%) [26]. The number of owner-reported health and behaviour problems decreased the likelihood of owners being certain they would acquire the same breed again, dropping to 20% for one problem reported and 12% for two problems [26].

Somewhat paradoxically, owners who considered their dog to be healthier than average for the breed had a reduced odds of reacquisition desire or of recommending their breed compared to those considered to have average health. This result may be influenced by skewed perceptions of what is ‘average’ for brachycephalic breeds. In this study population, previously published analyses indicated that 63.1% of owners considered their dog more healthy than average, with only 30.1% considered average, and just 6.8% less healthy than average [32]. As such, this variable is unlikely to be an accurate reflection of health status and requires further investigation. It is possible however that owners who considered their dog to be healthier than average are aware of health issues in their breed and worry they might be less ‘lucky’ if acquiring the same breed in the future, thus avoiding taking this chance.

Concerns over the welfare costs of breeding practices associated with producing brachycephalic dogs were rarely cited as a reason to recommend against their breed, with very few owners expressing guilt regarding acquiring breeds that they acknowledged to have common and severe health problems. This may be driven by processes of cognitive dissonance, when a person’s behaviour or belief is inconsistent with another belief they hold, resulting in one of the beliefs being modified in an attempt to reduce the dissonance [67]. Brachycephalic owners who love their dogs may be unwilling or unable to accept they had any role in their dog’s health problems by selecting a high-risk breed, or of promoting wider problems at a population level by increasing demand for brachycephalic breeds. Concerns over the expense associated with poor health were more common in this population, and it is possible that more targeted campaigning on specific undesirable elements of brachycephalic ownership, such as the potential financial burden, may avoid inciting further dissonance in owners who have emotional bonds with these breeds.

### Influence of the dog-owner relationship

Two positive facets of the dog-owner relationship (as quantified by the MDORS) increased the odds of reacquisition desire and recommendation; an increased level of emotional closeness, and a reduced perception of the costs of ownership. In a population of Danish dog owners, the dog-owner relationship (as quantified by the Lexington Attachment to Pets Scale [68]) predicted the likelihood of an owner being certain they would re-acquire a French Bulldog or a Cairn Terrier, but not a Cavalier King Charles Spaniel or Chihuahua [26]. It has previously been demonstrated that brachycephalic dog owners, particularly females without children in the household, form strong emotional bonds with their dogs [32]. This relationship may, to some extent, reflect a caregiver-infant relationship of owners seeking ‘perpetual children’ [69]. Indeed, some owners in the current study relished the dependency their dog had upon them and did not consider caring for their additional needs as burdensome. As such, actual level of care given, and perceived costs of ownership are likely to become uncoupled in this population. High levels of caregiving are associated with high levels of attachment to dogs; caregiving activities may facilitate attachment development, which then motivates sustained caregiving [65]. This may shape an owner’s ‘blueprint’ of dog ownership, which they may wish to emulate with future dogs they own. Longitudinal studies are required to evaluate the development of dog-owner attachment and its influence on dog breed-loyalty [28], for example, whether this ‘continued allegiance’ stems from initial or later ownership experiences, is a latent expression of childhood relationships with dogs, and crucially, whether breed-loyalty can be attenuated once developed.

### Influence of owner demographics

First-time dog owners had over twice the odds of reacquisition desire, and three times the odds of recommending their breed compared to owners who had owned any other dogs before. This effect may be explained by differences in what first-time owners vs. more experienced dog owners consider ‘normal’. If a person’s first ownership experience is with one of the studied brachycephalic breeds, their baseline for canine health and behaviour may become skewed. Signs of poor health may be considered ‘normal’ [70], and positive behavioural traits may be considered unique to their breed, even if available more widely. Combined, this may lead to more positive perceptions of their breed, increasing their desire to continue ownership of this breed and to promote them to others. Previous studies have identified that brachycephalic dog owners are more likely to be owning their breed for the first-time and be of a younger age, compared to owners of non-brachycephalic breeds [22], and thus this ownership group are likely key for future behaviour change initiatives around brachycephalic health. We did not explore the views of owners in this study who had previously owned dogs but were owning a brachycephalic breed for the first time. As these owners may have experience of dogs with fewer conformation-related disorders, their experiences and perceptions of brachycephalic breeds is of interest for future research.

## Limitations

Limitations of this study include the sampling strategy used and resulting study population. The sample was biased towards young Bulldogs, French Bulldogs and Pugs, which is likely due to the huge expansion in popularity of these breeds (particularly the latter two) in recent years, resulting in the rate of addition of new puppies to these breeds’ demographic profiles being much higher relative to other breeds that are not expanding. This bias towards younger dogs may also be due to the reduced life expectancy in extreme brachycephalic breeds (including the breeds studied here) compared to moderately brachycephalic and non-brachycephalic breeds (median 8.6 vs. 12.7 years), thus older dogs being less represented in studies such as this [5]. It is possible that owners of older dogs would have different views on reacquiring or recommending their breed. Although age of dog was a significant factor at the univariable level, it was non-significant in multivariable models after accounting for other factors. In addition, the use of a convenience sample may bias towards owners with certain beliefs or demographic characteristics, and the use of data from dogs and owners from different countries may complicate the generalisability of these results. A comparator population was not included in this study and thus further research is required to compare Bulldogs, French Bulldogs and Pugs with less popular brachycephalic breeds (e.g. Pekingese, Boston Terrier) and non-brachycephalic breeds.

## Future directions

These novel results offer important insights into common reasons explaining why owners would re-acquire or recommend brachycephalic breeds and can be used to inform interventions aimed at attenuating these desires. We hypothesise that differing factors may influence individual owners and/or would-be owners e.g. high hair-shedding may deter a house-proud owner, whereas high veterinary costs may deter an owner facing financial hardship. Determining the most effective intervention(s) requires further work to devise wider-scale initiatives based on theories from human behaviour change science, which are increasingly being adopted to tackle companion animal welfare issues [71, 72]. What is clear from the current paper is that ownership decision-making is a complex process and therefore the issues around ill-advised breed selection are not likely to be solved with simple solutions. Multi-stakeholder activities with consistent public messaging are likely to be integral to changing public opinion on the desirability of ownership of key challenged breeds [61, 73].

In the current study, we adopted a high quantity free-text approach for our qualitative data collection as we were motivated to identify common viewpoints, but consequently our data were relatively shallow. More in-depth qualitative research techniques e.g. semi-structured interviewing may be valuable in future research, for example, to glean deeper insights into the psychological processes underlying behaviour change e.g. identifying factors that changed the minds of owners who were contemplating buying a brachycephalic breed but did not, or owners who previously owned brachycephalic breeds but have chosen not to going forward.

## Conclusion

Owners of Bulldogs, French Bulldogs and Pugs dogs are highly likely to want to own their breed again in the future, and to recommend their breed to other owners. The development of breed-loyalty towards brachycephalic breeds may lead to their continued proliferation, and perpetuation of a high level of popularity, despite their substantial health risks. Although owners are initially attracted to the distinctive appearance of brachycephalic dogs, perceived breed-related behavioural traits are a core component of why owners perceive their breed positively, alongside the strong emotional bonds they inspire and perceived suitability for sedentary lifestyles. Understanding how breed-loyalty develops, and whether it can be attenuated will be key to controlling the current population boom in brachycephalic breeds in the long term. Although further work is required to devise effective larger-scale behaviour change interventions to attenuate the desire to reacquire and recommend brachycephalic breeds, in the short-term, these novel findings offer authentic accounts from existing owners that greatly increase our understanding of this pressing phenomenon and can be used to inform discussions by veterinarians and other animal health professionals when counselling would-be and current owners on breed selection.

## Supporting information

S1 File (DOCX)
