# Supplementary material for: Come for the looks, stay for the personality? A mixed methods investigation of reacquisition and owner recommendation of Bulldogs, French Bulldogs and Pugs
Source: PLoS One. 2020 Aug 26;15(8):e0237276. doi: 10.1371/journal.pone.0237276 (PMC7449392; doi:10.1371/journal.pone.0237276)
Supplement: S1 File — (DOCX) [file pone.0237276.s001.docx]

**Supplementary File 1**

Table S1. Univariable statistical associations between owner recommendation of their breed (binomial; Yes/No) and dog signalment, owner, dog health, owner expectations and dog-owner bond variables

| **Group** | **Variable** | **Sub-category** | **n** | **Recommend?** | | **OR**  **(95% CI)** | **P** |
| --- | --- | --- | --- | --- | --- | --- | --- |
|  |  |  |  | **No** | **Yes** |  |  |
| Dog demo-graphics | Breed | Bulldog | 638 | 43.8 | 56.2 | 0.46 (0.36-0.58) | <0.001 |
|  |  | French Bulldog | 741 | 35.4 | 64.6 | 0.65 (0.52-0.82) | <0.001 |
|  |  | Pug | 789 | 26.3 | 73.7 | *Reference* | |
|  | Age (years) | - | 2168 | 2.54 | 2.00 | 0.95 (0.91-0.98) | 0.001 |
| Owner demo-graphics | Gender | Female | 1929 | 35.6 | 64.4 | 0.62 (0.45-0.86) | 0.003 |
|  |  | Male | 230 | 25.6 | 74.4 | *Reference* | |
|  | Age category | 18-34 | 989 | 31.7 | 68.3 | 1.52 (1.08-2.13) | 0.016 |
|  |  | 35-54 | 983 | 36.0 | 64.0 | 1.25 (0.89-1.76) | 0.191 |
|  |  | 55-74 | 196 | 41.3 | 58.7 | *Reference* | |
|  | First-time dog owner | Yes | 607 | 40.5 | 59.5 | 2.85 (2.25-3.61) | <0.001 |
|  |  | No | 1553 | 19.3 | 80.7 | *Reference* | |
|  | Children in household | Yes | 989 | 35.0 | 65.0 | 1.05 (0.87-1.27) | 0.612 |
|  |  | No | 1177 | 33.9 | 66.1 | *Reference* | |
| Veterinary experiences | Vet costs / year  (£) | <250 | 1082 | 29.4 | 70.6 | *Reference* | |
|  |  | 250-499 | 340 | 32.3 | 67.7 | 0.87 (0.66-1.15) | 0.329 |
|  |  | 500-999 | 279 | 39.5 | 60.5 | 0.64 (0.47-0.85) | 0.002 |
|  |  | 1000-1499 | 121 | 48.1 | 51.9 | 0.45 (0.30-0.67) | <0.001 |
|  |  | 1500-1999 | 53 | 42.6 | 57.4 | 0.56 (0.31-1.02) | 0.057 |
|  |  | >2000 | 188 | 53.5 | 46.5 | 0.36 (0.26-0.50) | <0.001 |
|  | Vet costs to date  (£) | <250 | 623 | 27.4 | 72.6 | *Reference* | |
|  |  | 250-499 | 352 | 27.7 | 72.3 | 0.99 (0.73-1.34) | 0.942 |
|  |  | 500-999 | 301 | 33.3 | 66.7 | 0.76 (0.55-1.04) | 0.082 |
|  |  | 1000-1499 | 171 | 35.5 | 64.5 | 0.69 (0.47-1.01) | 0.052 |
|  |  | 1500-1999 | 82 | 32.9 | 67.1 | 0.77 (0.45-1.30) | 0.332 |
|  |  | >2000 | 545 | 49.2 | 50.8 | 0.39 (0.30-0.51) | <0.001 |
|  | No. of conformation- related surgeries | - | 2168 | 0 (0-1) | 0 (0-0) | 0.64 (0.56-0.72) | <0.001 |
| Health scores | ORB | - | 2168 | 7 (4-12) | 6 (3-10) | 0.92 (0.75-0.98) | <0.001 |
|  | Heat intolerance | - | 2168 | 3 (2-5) | 3 (2-4) | 0.87 (0.84-0.91) | <0.001 |
|  | Eating difficulty | - | 2168 | 1 (0-3) | 1 (0-2) | 0.92 (0.88-0.95) | <0.001 |
|  | Disordered sleep | - | 2168 | 6 (2-10) | 6 (2-9) | 0.99 (0.98-1.01) | 0.580 |
| Health perceptions | No. of BOAS-related health problems | - | 2168 | 1 (0-1) | 0 (0-1) | 0.63 (0.57-0.70) | <0.001 |
|  | Compared to the rest of their breed | Much less healthy | 31 | 67.7 | 32.3 | 0.23 (0.11-0.50) | <0.001 |
|  |  | Less healthy | 99 | 57.6 | 42.4 | 0.36 (0.23-0.55) | <0.001 |
|  |  | Average health | 577 | 32.8 | 67.2 | *Reference* | |
|  |  | Healthier | 783 | 32.8 | 67.2 | 0.99 (0.79-1.25) | 0.979 |
|  |  | Much healthier | 456 | 32.2 | 67.8 | 1.02 (0.79-1.33) | 0.860 |
|  | Overall health rating | Worst possible | 4 | 25.0 | 75.0 | 0.86 (0.09-8.31) | 0.894 |
|  |  | Very poor | 38 | 71.1 | 28.9 | 0.12 (0.06-0.24) | <0.001 |
|  |  | Moderately poor | 101 | 62.4 | 37.6 | 0.17 (0.11-0.27) | <0.001 |
|  |  | Good | 161 | 50.3 | 49.7 | 0.28 (0.20-0.41) | <0.001 |
|  |  | Moderately good | 251 | 42.2 | 57.8 | 0.39 (0.29-0.54) | <0.001 |
|  |  | Very good | 778 | 32.9 | 67.1 | 0.58 (0.46-0.74) | <0.001 |
|  |  | Best possible | 612 | 22.2 | 77.8 | *Reference* | |
| Expectations vs. reality | Veterinary costs | Less | 246 | 32.5 | 67.5 | 0.98 (0.73-1.31) | 0.892 |
|  |  | Met | 1250 | 32.1 | 67.9 | *Reference* | |
|  |  | More | 399 | 43.9 | 56.1 | 0.61 (0.48-0.76) | <0.001 |
|  | Exercise levels | Less | 188 | 51.6 | 48.4 | 0.48 (0.35-0.65) | <0.001 |
|  |  | Met | 1279 | 33.9 | 66.1 | *Reference* | |
|  |  | More | 470 | 29.1 | 70.9 | 1.24 (0.99-1.57) | 0.063 |
|  | Overall behaviour | Better | 412 | 28.2 | 71.8 | 1.32 (1.04-1.69) | 0.025 |
|  |  | Met | 1287 | 34.1 | 65.9 | *Reference* | |
|  |  | Worse | 244 | 47.5 | 52.5 | 0.57 (0.43-0.75) | <0.001 |
|  | Maintenance | Less | 150 | 26.7 | 73.3 | 1.43 (0.98-2.09) | 0.060 |
|  |  | Met | 1596 | 34.3 | 65.7 | *Reference* | |
|  |  | More | 169 | 45.6 | 54.4 | 0.62 (0.45-0.86) | 0.004 |
| MDORS | Emotional closeness | - | 2168 | 4.4  (3.8-4.8) | 4.5  (3.9-4.8) | 1.36 (1.16-1.59) | <0.001 |
|  | Perceived costs | - | 2168 | 4.2  (3.9-4.6) | 4.3  (4.0-4.8) | 1.59 (1.35-1.88) | <0.001 |
|  | Dog-owner interactions | - | 2168 | 4.1  (3.8-4.3) | 4.1  (3.9-4.4) | 1.19 (0.95-1.48) | 0.133 |

Table S2. Univariable statistical associations with owner reacquisition of their breed (binomial; Yes/No) and dog signalment, owner, dog health, owner expectations and dog-owner bond variables

| **Group** | **Variable** | **Sub-category** | **n** | **Reacquire?** | | **OR**  **(95% CI)** | **P** |
| --- | --- | --- | --- | --- | --- | --- | --- |
|  |  |  |  | **No** | **Yes** |  |  |
| Dog demo-graphics | Breed | Bulldog | 638 | 6.9 | 93.1 | 0.98 (0.64-1.48) | 0.904 |
|  |  | French Bulldog | 741 | 7.4 | 92.6 | 0.90 (0.61-1.33) | 0.590 |
|  |  | Pug | 789 | 6.7 | 93.3 | *Reference* | |
|  | Age (years) | - | 2168 | 3.0 | 2.08 | 0.93 (0.88-0.98) | 0.009 |
| Owner demo-graphics | Gender | Female | 1929 | 7.3 | 92.7 | 0.64 (0.34-1.20) | 0.163 |
|  |  | Male | 230 | 4.8 | 95.2 | *Reference* | |
|  | Age category | 18-34 | 989 | 7.0 | 93.0 | 1.11 (0.62-1.98) | 0.725 |
|  |  | 35-54 | 983 | 6.9 | 93.1 | 1.12 (0.63-2.00) | 0.705 |
|  |  | 55-74 | 196 | 7.7 | 92.3 | *Reference* | |
|  | First-time dog owner | Yes | 607 | 4.1 | 95.9 | 2.06 (1.33-3.20) | <0.001 |
|  |  | No | 1553 | 8.1 | 91.9 | *Reference* | |
|  | Children in household | Yes | 989 | 6.8 | 93.2 | 1.06 (0.76-1.48) | 0.743 |
|  |  | No | 1177 | 7.1 | 92.9 | *Reference* | |
| Veterinary experiences | Vet costs / year  (£) | <250 | 1082 | 4.7 | 95.3 | *Reference* | |
|  |  | 250-499 | 340 | 5.6 | 94.4 | 0.84 (0.49-1.44) | 0.523 |
|  |  | 500-999 | 279 | 7.9 | 92.1 | 0.58 (0.34-0.97) | 0.038 |
|  |  | 1000-1499 | 121 | 13.2 | 86.6 | 0.33 (0.18-0.59) | <0.001 |
|  |  | 1500-1999 | 53 | 9.4 | 90.6 | 0.48 (0.18-1.25) | 0.131 |
|  |  | >2000 | 188 | 14.9 | 85.1 | 0.28 (0.17-0.46) | <0.001 |
|  | Vet costs to date  (£) | <250 | 623 | 3.7 | 96.3 | *Reference* | |
|  |  | 250-499 | 352 | 5.7 | 94.3 | 0.63 (0.34-1.17) | 0.145 |
|  |  | 500-999 | 301 | 6.6 | 93.4 | 0.54 (0.29-0.99) | 0.049 |
|  |  | 1000-1499 | 171 | 4.7 | 95.3 | 0.78 (0.34-1.78) | 0.559 |
|  |  | 1500-1999 | 82 | 4.9 | 95.1 | 0.75 (0.25-2.22) | 0.602 |
|  |  | >2000 | 545 | 12.5 | 87.5 | 0.27 (0.17-0.44) | <0.001 |
|  | No. of conformation- related surgeries | - | 2168 | 0 (0-1) | 0 (0-0) | 0.64 (0.56-0.74) | <0.001 |
| Health scores | ORB | - | 2168 | 10 (5-16.5) | 6 (3-10) | 0.91 (0.89-0.93) | <0.001 |
|  | Heat intolerance | - | 2168 | 5 (3-7) | 3 (2-4) | 0.72 (0.67-0.77) | <0.001 |
|  | Eating difficulty | - | 2168 | 1 (0-3.5) | 1 (0-2) | 0.89 (0.84-0.93) | <0.001 |
|  | Disordered sleep | - | 2168 | 7 (3-10) | 6 (2-9) | 0.96 (0.93-0.99) | 0.020 |
| Health perceptions | No. of BOAS-related health problems | - | 2168 | 1 (1-2) | 0 (0-1) | 0.43 (0.37-0.50) | <0.001 |
|  | Compared to the rest of their breed | Much less healthy | 31 | 34.4 | 65.6 | 0.16 (0.07-0.34) | <0.001 |
|  |  | Less healthy | 99 | 23.5 | 76.5 | 0.26 (0.16-0.45) | <0.001 |
|  |  | Average health | 577 | 7.5 | 92.5 | *Reference* | |
|  |  | Healthier | 783 | 4.3 | 95.7 | 1.79 (1.16-2.77) | 0.009 |
|  |  | Much healthier | 456 | 5.5 | 94.5 | 1.39 (0.85-2.25) | 0.188 |
|  | Overall health rating | Worst possible | 4 | 40.0 | 60.0 | 0.04 (0.01-0.23) | <0.001 |
|  |  | Very poor | 38 | 43.9 | 56.1 | 0.03 (0.01-0.07) | <0.001 |
|  |  | Moderately poor | 101 | 22.8 | 77.2 | 0.08 (0.04-0.16) | <0.001 |
|  |  | Good | 161 | 14.8 | 85.2 | 0.14 (0.07-0.26) | <0.001 |
|  |  | Moderately good | 251 | 8.7 | 91.3 | 0.25 (0.13-0.48) | <0.001 |
|  |  | Very good | 778 | 4.4 | 95.6 | 0.51 (0.28-0.94) | 0.031 |
|  |  | Best possible | 612 | 2.3 | 97.7 | *Reference* | |
| Expectations vs. reality | Veterinary costs | Less | 246 | 5.9 | 94.1 | 0.76 (0.43-1.34) | 0.338 |
|  |  | Met | 1250 | 4.5 | 95.5 | *Reference* | |
|  |  | More | 399 | 14.9 | 85.1 | 0.27 (1.90-0.39) | <0.001 |
|  | Exercise levels | Less | 188 | 21.0 | 79.0 | 0.23 (0.16-0.35) | <0.001 |
|  |  | Met | 1279 | 5.8 | 94.2 | *Reference* | |
|  |  | More | 470 | 4.4 | 95.6 | 1.34 (0.84-2.15) | 0.225 |
|  | Overall behaviour | Better | 412 | 4.6 | 95.4 | 1.46 (0.90-2.36) | 0.129 |
|  |  | Met | 1287 | 6.6 | 93.4 | *Reference* | |
|  |  | Worse | 244 | 13.7 | 86.3 | 0.44 (0.29-0.67) | <0.001 |
|  | Maintenance | Less | 150 | 6.8 | 93.2 | 0.91 (0.49-1.69) | 0.772 |
|  |  | Met | 1596 | 6.2 | 93.8 | *Reference* | |
|  |  | More | 169 | 14.9 | 85.1 | 0.38 (0.24-0.58) | <0.001 |
| MDORS | Emotional closeness | - | 2168 | 3.9  (3.4-4.6) | 4.5  (3.9-4.8) | 2.47 (1.91-3.20) | <0.001 |
|  | Perceived costs | - | 2168 | 3.9  (3.5-4.3) | 4.3  (4.0-4.7) | 2.98 (2.32-3.82) | <0.001 |
|  | Dog-owner interactions | - | 2168 | 4.0  (3.7-4.3) | 4.1  (3.9-4.3) | 2.06 (1.44-2.96) | <0.001 |
